# Cost‐Effectiveness and Budget Impact Analysis of the Implementation of Differentiated Service Delivery Models for HIV Treatment in Mozambique: a Modelling Study

**DOI:** 10.1002/jia2.26275

**Published:** 2024-05-27

**Authors:** Dorlim Antonio Moiana Uetela, Marita Zimmermann, Sérgio Chicumbe, Eduardo Samo Gudo, Ruanne Barnabas, Onei Andre Uetela, Aneth Dinis, Orvalho Augusto, Sandra Gaveta, Aleny Couto, Irénio Gaspar, Hélder Macul, James P. Hughes, Sarah Gimbel, Kenneth Sherr

**Affiliations:** ^1^ Instituto Nacional de Saúde Marracuene Mozambique; ^2^ Department of Global Health University of Washington Seattle Washington USA; ^3^ The Comparative Health Outcomes, Policy, and Economics Institute University of Washington Seattle Washington USA; ^4^ Division of Infectious Diseases Massachusetts General Hospital Harvard Medical School Boston Massachusetts USA; ^5^ National STI‐HIV/AIDS Program Ministry of Health Maputo Mozambique; ^6^ School of Public Health–Biostatistics University of Washington Seattle Washington USA; ^7^ Department of Child Family and Population Health Nursing University of Washington Seattle Washington USA; ^8^ Department of Epidemiology University of Washington Seattle Washington USA; ^9^ Department of Industrial and Systems Engineering University of Washington Seattle Washington USA

**Keywords:** HIV, differentiated service delivery, cost‐effectiveness analysis, budget impact analysis, modelling, Mozambique

## Abstract

**Introduction:**

In 2018, the Mozambique Ministry of Health launched guidelines for implementing differentiated service delivery models (DSDMs) to optimize HIV service delivery, improve retention in care, and ultimately reduce HIV‐associated mortality. The models were fast‐track, 3‐month antiretrovirals dispensing, community antiretroviral therapy groups, adherence clubs, family approach and three one‐stop shop models: adolescent‐friendly health services, maternal and child health, and tuberculosis. We conducted a cost‐effectiveness analysis and budget impact analysis to compare these models to conventional services.

**Methods:**

We constructed a decision tree model based on the percentage of enrolment in each model and the probability of the outcome (12‐month retention in treatment) for each year of the study period—three for the cost‐effectiveness analysis (2019–2021) and three for the budget impact analysis (2022–2024). Costs for these analyses were primarily estimated per client‐year from the health system perspective. A secondary cost‐effectiveness analysis was conducted from the societal perspective. Budget impact analysis costs included antiretrovirals, laboratory tests and service provision interactions. Cost‐effectiveness analysis additionally included start‐up, training and clients’ opportunity costs. Effectiveness was estimated using an uncontrolled interrupted time series analysis comparing the outcome before and after the implementation of the differentiated models. A one‐way sensitivity analysis was conducted to identify drivers of uncertainty.

**Results:**

After implementation of the DSDMs, there was a mean increase of 14.9 percentage points (95% CI: 12.2, 17.8) in 12‐month retention, from 47.6% (95% CI, 44.9–50.2) to 62.5% (95% CI, 60.9–64.1). The mean cost difference comparing DSDMs and conventional care was US$ –6 million (173,391,277 vs. 179,461,668) and –32.5 million (394,705,618 vs. 433,232,289) from the health system and the societal perspective, respectively. Therefore, DSDMs dominated conventional care. Results were most sensitive to conventional care interaction costs in the one‐way sensitivity analysis. For a population of 1.5 million, the base‐case 3‐year financial costs associated with the DSDMs was US$550 million, compared with US$564 million for conventional care.

**Conclusions:**

DSDMs were less expensive and more effective in retaining clients 12 months after antiretroviral therapy initiation and were estimated to save approximately US$14 million for the health system from 2022 to 2024.

## 1 INTRODUCTION

In November 2018, Mozambique's Ministry of Health (MISAU) launched guidelines for the nationwide implementation of eight differentiated service delivery models (DSDMs) to increase efficiency and reduce HIV‐associated mortality. The eight models were: (1) adherence clubs (ACs); (2) community antiretroviral therapy groups (CAGs); (3) family approach (FA); (4) fast‐track (FT); (5) one‐stop shop (OSS) for adolescent‐friendly health services (OSS‐AFHS); (6) OSS for maternal and child health services (OSS‐MCH); (7) one‐stop shop for tuberculosis services (OSS‐TB);[1, 2] and (8) 3‐month antiretrovirals dispensing (3MMD). They were expected to be economically advantageous for the health system and its clients as they reduce visit frequency for clients established on antiretroviral therapy (ART) and integrate services for clients using other services at the health facility, thus minimizing unnecessary visits [3].

The body of literature on the economic evaluation of DSDMs is growing in sub‐Saharan Africa. However, assessing their overall economic impact is difficult because of the specific costs of DSDM and conventional care in each country, and inconsistent results in health outcomes [4, 5]. Studies have reported DSDMs being both cost‐saving and more expensive, depending on the model and the conventional care comparator. For example, a review including several African countries found that costs varied from 11.4% less to 9.2% more than conventional care [6]. As such, the economic impact of each model needs to be assessed in a country‐specific context [4, 5, 7].

We conducted a cost‐effectiveness analysis (CEA) and a budget impact analysis (BIA) of the implementation of the eight models in Mozambique to inform MISAU of their economic impact.

## 2 METHODS

### 2.1 Setting

In Mozambique, MISAU is the only official HIV service provider, offering both DSDMs and conventional care for HIV treatment free of charge. Eligible clients can choose to be enrolled in either care services, with no negative consequences to their care. The eligibility criterion depend on the DSDM. For AC, CAG, FT and 3MMD, the criterion are (1) enrolment on ART for more than 6 months, (2) viral suppression and (3) no opportunistic infections. For the OSS models, the criterium is simultaneous enrolment on ART and on the services where the OSS models are offered.

### 2.2 Conventional and differentiated service delivery models

Conventional care consists of monthly appointments for clinical observation, medication prescription and dispensation. Laboratory tests include haemoglobin, creatinine, alanine aminotransferase and CD4 count performed semi‐annually, and viral load measurements performed at 6 and 12 months after ART initiation and annually thereafter. The intervention is a package of the eight DSDMs and conventional care, as described in detail elsewhere [8].

### 2.3 Cost‐effectiveness and budget impact analyses

The outcome of interest for both the CEA and BIA was 12‐month retention after ART initiation, comparing DSDMs implementation period to a counterfactual scenario (i.e. if the DSDMs had not been implemented). The CEA was conducted primarily from MISAU's perspective and secondarily from the societal perspective, considering the 3 years of DSDMS implementation (2019−2021) as the time horizon. The BIA was conducted from MISAU's perspective over a 3‐year period (2022−2024).

#### 2.3.1 The model

**Figure 1:**
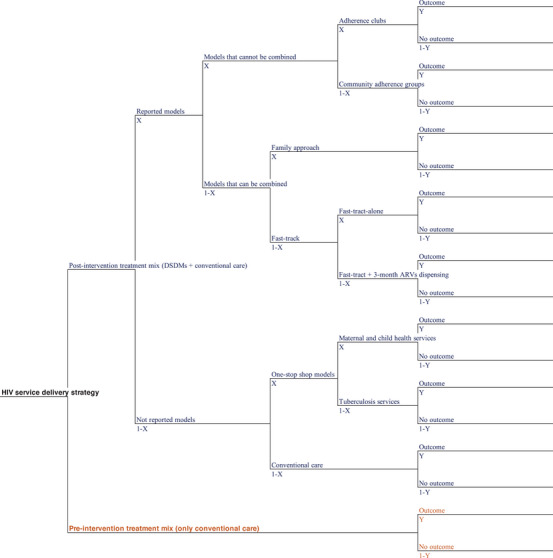
Decision tree model. X, branch probability; Y, outcome probability.

We constructed a decision tree model (Figure 1) for each year of the study period based on the estimated percentage of enrolment in each treatment model.

The percentage of enrolment in each DSDM was estimated based on data from the country's President's Emergency Plan for AIDS Relief (PEPFAR) monthly monitoring database (Analyzing Joint Underperformance and Determining Assistance—AJUDA) for the models reported (AC, CAG, FA, FT and 3MMD) and from MISAU's HIV/AIDS national programme annual reports for the OSS‐MCH and OSS‐TB [9]. Data on the OSS‐AFHS model are not available; therefore, clients enrolled in this model were counted in the conventional model. The percentage of clients enrolled in the conventional is also not reported; we estimated it by calculating the difference between the total number of clients enrolled in ART and those enrolled in at least one DSDM.

In the AJUDA database, the sum of the reported number of clients enrolled in each model is more than the reported number of clients enrolled in at least one model, because a client can be enrolled in more than one DSDM (for models that can be combined). However, each model is reported individually, resulting in duplicate reporting for the combined models. As there are no data on which DSDMs are being combined and double reported, the percentages of enrolment in each DSDM were estimated as follows:

1. AC and CAG cannot be combined with other DSDMs; therefore, we assumed no duplication on the reported data and estimated the percentage of enrolment based on these numbers.
2. To estimate the percentage of enrolment in FT alone and FA, we subtracted the number reported for FA from the number reported for FT. Thus, duplications would conservatively be counted only in FA, under the assumption that FA is less economically advantageous than FT, due to more clinical visits.
3. To estimate the percentage of enrolment in FT combined with 3MMD (3MMD must always be combined with FT), we subtracted the estimated numbers of enrolment in AC, CAG, FA and FT alone from the reported number of clients enrolled in at least one model.

For OSS‐MCH and OSS‐TB, we used data from MISAU's HIV/AIDS national programme annual reports. Percentages of enrolment were calculated based on the reported numbers, given that all patients enrolled in ART in these services are assumed to be in the respective OSS model.

#### 2.3.2 Cost estimation

For the CEA, we primarily estimated the economic costs from MISAU's perspective and secondarily from the societal perspective. For the BIA, financial costs were estimated from MISAU's perspective. Economic costs are all explicit and implicit costs related to the resources utilized and foregone in a decision, while financial costs are specifically monetary expenses [10]. Costs were estimated for DSDMs and conventional care per client‐year. For the BIA, they included antiretrovirals, laboratory tests and service provision interactions (clinical, pharmacy and group interactions). For the CEA from MISAU's perspective, the costs for conventional care were the same as for the BIA. For DSDM, costs additionally included DSDMs start‐up and training. From the societal perspective, clients’ opportunity costs were added to the costs from MISAU's perspective.

Antiretrovirals costs were obtained from MISAU's HIV/AIDS national programme. Laboratory test costs were obtained from the National Institute of Health Laboratories. Service provision interactions for both conventional care and DSDMs were estimated based on a literature review of HIV treatment in sub‐Saharan Africa. The conventional care costs included infrastructure use, human resources and overhead. DSDM costs included specific interactions or activities in addition to, or substitution of, the conventional model interactions, according to specific model characteristics.

To calculate the cost of each conventional care interaction, we compared the costs and services offered at conventional care interactions described in other studies [4, 5, 11, 12, 13, 14] and only included the costs applicable to the Mozambique context. When only annual costs were presented, besides the services offered, we also considered the number of interactions offered in the model of care in those studies to estimate the costs per interaction.

To calculate the cost per person per each AC meeting, we included the costs applicable to the Mozambique context based on the breakdown of costs found in the literature [4, 11, 15]. These included (1) nurse time, (2) pharmacy technician time, (3) club facilitators time, (4) ongoing club mentorship, and (5) infrastructure and overheads. The total cost of visits per person‐year for clients using AC was calculated by multiplying the cost per club meeting by four (the number of club meetings per year). CAG interactions costs per person were estimated from the literature [13, 15, 16, 17]. To calculate the cost per person‐year for CAG, we included costs for two conventional care interactions (required for clinical and laboratory follow‐up) and 10 CAG interactions.

We considered the cost of each clinical and pharmacy interaction for FA and FT to be equal to the conventional model, and we added group model management for the FA model. The total cost of these interactions per person‐year for FA was calculated by adding the cost for group model management to the clinical and pharmacy interaction costs multiplied by 12 (the number of interactions per year). For FT, the total cost per person‐year of clinical interaction was calculated by multiplying the cost per interaction by 2 (the number of interactions per year), and of pharmacy interactions was calculated by multiplying the cost per interactions by 4 or by 12, depending on whether the FT was combined or not with the 3MMD.

The economic and financial costs of the models described above were assumed to be the same. The economic costs for each interaction of OSS‐MCH and OSS‐TB for the CEA were estimated as described for AC interactions, given that the services offered in these models’ interactions are the same as in the AC interactions. To determine the financial costs of the OSS model interactions for the BIA, we considered it to be half of the estimated economic costs, assuming that the other half of the costs would be covered by the primary services (MCH and TB) used by the clients.

DSDM start‐up costs (guideline development and distribution, demand generation activities and initial training), and ongoing training costs were estimated based on the information provided by MISAU's HIV/AIDS national programme. These costs were only considered for the DSDMs, given that the conventional care is established in Mozambique since 2004 and no additional costs were incurred in these categories during the period of analysis.

Clients’ opportunity costs included travel time to the health facility and time spent at the health facility for service provision. The total opportunity cost per conventional care interaction was estimated as a half‐day of wages, based on the country's average wage, considering on average 1 hour of travel time and 3 hours of time spent at the health facility [18, 19]. Medication pick‐ups for the 3MMD were estimated to be 1.5 hours, AC interactions and CAG interactions in the community were estimated to be 3.5 and 1 hour, respectively. For FA, we considered half of the opportunity costs of the conventional visit, assuming that clients using this model reduce their visits by half because members of the same household are scheduled to the same day [18, 19, 20]. Opportunity costs were estimated in Mozambique metical and converted to US dollars (US$) using the 2021 annual average exchange rate of 63.25 metical per US$1. All other costs were estimated in US$, and no discount was applied. Costs were estimated for each year from 2019 to 2021 and assumed to be constant from 2021 to 2024.

The total cost per year per model of service delivery (conventional or DSDM) was estimated considering the expected number interactions for each model of service, based on their estimated retention in treatment. For convenience, we assumed that clients not retained in treatment stopped it in the middle of the year.

#### 2.3.3 Effectiveness

Effectiveness, measured as retention at 12 months after ART initiation, was estimated by comparing the periods before and after DSDMs implementation, as described in the DSDMs impact study [21]. We applied an uncontrolled interrupted time series design to compare the outcome before and after implementation of the DSDMs nationwide, using data from all clients enrolled in HIV treatment from January 2016 to June 2020 (i.e. follow‐up data through June 2021) in health facilities using the electronic patient tracking system, which feeds the Mozambique Antiretroviral Therapy (MozART) database [22].

#### 2.3.4 Sensitivity analysis

We conducted a one‐way sensitivity analysis by inputting the lower and higher estimated costs for the following categories: (1) conventional service interactions; (2) AC interactions; (3) CAG interactions; (4) antiretrovirals; (5) laboratory tests; (6) start‐up and training; and (7) opportunity costs. Interactions for other models were not included because their costs were estimated based on conventional, AC or CAG interactions. Table 1 shows the models’ inputs.

**Table 1:** Cost‐effectiveness and budget impact analysis models inputs by study period

	Value (range) by year		
Data inputs	2019	2020	2021−2024
**Percentage of enrolment**			
Conventional care	49.7	19.9	20
Adherence club	0.2	0.2	0.2
Community adherence group	8.7	9	8.9
Family approach	1.8	3.3	3.7
Fast‐track alone	20.8	34	38
Fast‐track combined with 3MMD	11.8	22.2	19.3
One‐stop shop MCH	5.4	9.2	8.9
One‐stop shop TB	1.5	2.2	1.8
**Population size**	1,338,100	1,402,902	1,535,575
**Client opportunity cost per hour (US$)**	1.6 (0.8−2.4)	1.8 (0.9−2.6)	1.2 (0.6−1.7) [23]
**Costs for the health system (US$)**	**2019**−**2024**		
Antiretrovirals per year (first line)	43 (38.7−47.3)		
Laboratory tests per year	64 (57.6−70.4)		
Conventional care clinical interaction^a^	4.47 (1.11−10.32) [4, 11, 12, 13, 14]		
Conventional care pharmacy interaction^a^	1.12 (0.28−5.58) [5, 11, 16, 17, 18]		
Adherence club and one‐stop shop MCH and TB interactions^b^	6.67 (5.87−16.52) [4, 11, 15]		
Community adherence group interactions	2.77 (1.37−9.8) [13, 15, 16, 17]		
DSDMs coordination for family approach visits^c^	0.4 (0.26−0.53)		
Start‐up and training costs	383,466.67 (345,120.00−421,813.33)		

Abbreviations: 3MMD, 3‐month antiretrovirals dispensing; DSDMs, differentiated service delivery models; MCH, maternal and child health; TB, tuberculosis.

^a^ Conventional care visits to the health facility include clinical observations, antiretroviral therapy support, and medication prescription and pick‐up at the health facility pharmacy. These visits were divided into clinical and pharmacy interactions to simplify the cost estimation of the models family approach, fast‐track, 3‐month antiretrovirals dispensing, which were based on comparative activities with conventional care.

^b^ For the budget impact analysis, the costs for one‐stop shop MCH and TB interactions were considered half of the economic cost, assuming that the other half was covered by the primary services used by clients in these models.

^c^ Estimated from costs breakdown for adherence clubs and community adherence groups.

### 2.4 Ethics

This work was approved by the Mozambique National Ethics Committee (634/CNBS/20) and the University of Washington Institutional Review Board (FWA#00006878). MISAU gave administrative approval to use client data (1984/GMS/002/2020). Client informed consent was not required by the Mozambique National Ethics Committee as the study is a secondary analysis of routine programme data without any identifying information and a literature review.

## 3 RESULTS

### 3.1 Cost‐effectiveness analysis

#### 3.1.1 Costs

From MISAU's perspective, the estimated economic costs per person‐year for each model, including antiretrovirals and laboratory tests costs, from the most to the least expensive, were US$187 for OSS‐MCH and OSS‐TB, US$ 179 for FA, US$174 for conventional care, US$144 for CAG, US$134 for AC, US$129 for FT alone and US$120 for FT combined with 3MMD. From the societal perspective, the costs were US$251 for conventional care, US$245 for OSS‐MCH and OSS‐TB, US$218 for FA, US$165 for CAG, US$162 for FT alone, US$153 for AC, and US$138 for FT and 3MMD.

We calculated the total costs for DSDMs and conventional care by category, and the cost difference between them by year, from the health system and societal perspectives (Table 2). The mean cost for DSDMs and conventional care from the health system perspective was US$173,391,277 and US$179,461,668, respectively. The main cost drivers for conventional care were service provision interactions followed by laboratory tests, while for DSDMs were laboratory tests followed by service provision interactions. The opportunity costs were US$221,314,340 and US$253,770,621 for DSDMs and conventional care, respectively.

**Table 2:** Total cost per year and study period for DSDMs and conventional care, by cost category

	2019 costs (US$)			2020 costs (US$)			2021 costs (US$)			Study period mean costs (US$)		
Cost category	DSDMs	Conventional	Difference	DSDMs	Conventional	Difference	DSDMs	Conventional	Difference	DSDMs	Conventional	Difference
**MISAU's perspective**												
Services interactions	51,647,955	67,336,021	−15,688,066	44,766,695	67,523,554	−22,756,860	49,717,206	62,722,026	−13,004,820	48,710,618	65,860,534	−17,149,915
ARVs	44,592,183	43,153,725	1,438,458	49,466,325	43,433,846	6,032,479	55,795,118	47,211,253	8,583,864	49,951,208	44,599,608	5,351,600
Lab tests	66,369,760	64,228,800	2,140,960	73,624,297	64,645,724	8,978,573	83,043,896	78,130,056	4,913,840	74,345,984	69,001,527	5,344,458
Start‐up and training	406,800	—	406,800	371,800	—	371,800	371,800	—	371,800	383,467	—	383,467
Total	163,016,697	174,718,546	−11,701,849	168,229,116	175,603,124	−7,374,008	188,928,019	188,063,335	864,684	173,391,277	179,461,668	−6,070,391
**Societal perspective**												
Clients’ opportunity costs	218,821,182	252,291,176	−33,469,994	218,509,418	260,589,602	−42,080,185	226,612,421	248,431,083	−21,818,662	221,314,340	253,770,621	−32,456,280
Total	381,837,879	427,009,722	−45,171,843	386,738,534	436,192,727	−49,454,193	415,540,440	436,494,418	−20,953,978	394,705,618	433,232,289	−38,526,671

Abbreviations: ARVs, antiretrovirals; DSDMs, differentiated service delivery models; MISAU, Mozambique Ministry of Health.

#### 3.1.2 Effectiveness

Table 3 presents the effectiveness results of the implementation of DSDMs, calculated as the difference in the percentage points of the outcome comparing DSDMs and conventional care, per year of the study and the study period mean. Overall and by the year of the study, the DSDMs were more effective than conventional care in retaining clients 12 months after ART initiation.

**Table 3:** Effectiveness of DSDMs by study period

	12‐month retention in care % (95% CI)		Effectiveness of DSDMs
Year	DSDMs	Conventional	(95% CI)
2019	55.05% (53.08–57.00)	51.95% (49.92–53.99)	3.09% (0.55–5.62)
2020	64.05% (62.40–65.70)	46.67% (43.84–49.51)	17.37% (14.44–20.31)
2021	68.65% (66.56–70.55)	45.04% (40.78–47.29)	24.52% (21.06–27.98)
Study period (2019–2021) mean	62.55% (60.94–64.15)	47.55% (44.86–0.25)	14.99% (12.19–17.79)

Abbreviation: DSDMs, differentiated service delivery models.

#### 3.1.3 Incremental cost‐effectiveness ratio

Although the costs were higher for DSDMs comparing to conventional care in 2021, for the 3 years of the study period (2019–2021), the mean cost difference comparing DSDMs to conventional model of treatment was US$‐6,070,391 and US$‐38,526,671 from health system and societal perspective, respectively, and the mean effectiveness difference was 15%. The Incremental cost‐effectiveness ratio (ICER) was not calculated given that DSDMs were less expensive and more effective, thus dominating the conventional model of care during the evaluation period.

#### 3.1.4 Sensitivity analysis

**Figure 2:**
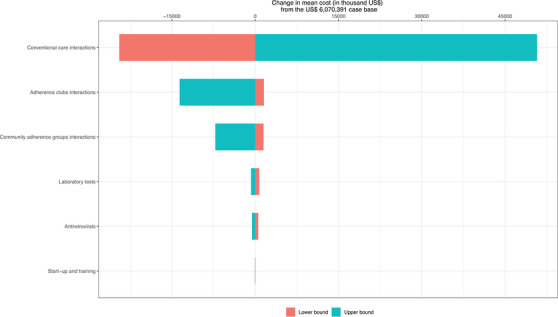
One‐way sensitivity analysis.

For the primary analysis, service provision interactions for the conventional care, and start‐up and training were the most and least influential cost category, respectively (Figure 2). For the secondary analysis, a sensitivity analysis of the opportunistic costs resulted in a change of US$+/–12,724,053 from the US$‐38,526,671 from the base case scenario. The detailed changes in the costs based on the higher and lower estimates of each of the included costs are presented in the Supplementary Material.

### 3.2 Budget impact analysis

Considering a population of 1,535,575 enrolled in HIV treatment in each year from 2022 to 2024, and the same treatment mix observed in 2021, we estimated an effectiveness of 24.5% and financial costs of US$550,118,496 and US$564,190,006 for DSDMs and conventional care, respectively. Other than the financial cost per person‐year for OSS‐MCH and OSS‐TB, which was estimated at US$147, the costs per person‐year for all other models, as well as the main drivers of the total costs, were the same as for the CEA.

## 4 DISCUSSION

Our study analyses the economic impact of the nationwide implementation of eight DSDMs in Mozambique, using a conservative approach to inform MISAU on the least expected benefit of implementing these models for both the health system and its clients, through a CEA and BIA. Our study findings revealed that DSDMs were less expensive and more effective in retaining clients 12 months after ART initiation, compared with conventional care. We estimated that the implementation of these models will result in savings of approximately US$14 million to the health system between 2022 and 2024.

Our main objective was to inform MISAU on the cost‐effectiveness and budget impact of DSDMs for informed decision‐making. However, given that one of the most acclaimed benefits of the implementation of the DSDMs is that they reduce the treatment burden to its clients, we also explored the societal perspective of this implementation by including two important treatment‐related costs—the travel time and the time spent at the health facility to access treatment services. The implementation of DSDMs during the 3 years of study was estimated to have saved US$6 million for the health system and US$32 million for its clients, supporting the theory that these models should be economically beneficial to the health system and its clients.

When comparing the cost of each model per person‐year to conventional care, from MISAU's perspective, the OSS‐MCH, OSS‐TB and FA models were more expensive, whereas CAG, AC, FT alone and FT combined with 3MMD were less expensive. The OSS models were the most expensive because they have the same number of interactions as conventional care plus the integration costs. However, this comparison does not account for the fact that OSS models offer more services than what is offered in the conventional care. FA was also estimated to be more expensive because of the additional costs of group model management, but it can be combined with other models with less service interactions, such as FT and 3MMD, resulting in lower costs than for FA alone. However, we used a conservative approach by assuming that clients using FA were not using other models. AC, CAG, FT alone and FT combined with 3MMD were less expensive than conventional care because they have fewer service provision interactions.

From the societal perspective, the conventional care was more expensive than all individual DSDMs because conventional service provision interactions are more frequent and longer in duration than interactions in DSDMs. FT, 3MMD, CAG and FA models save clients’ opportunity costs by reducing the frequency of visits to the health facility. FT and 3MMD increase visits spacing, CAG has monthly community meetings instead of health facility visits and FA benefits clients who are also caregivers by offering services for both caregivers and their dependents during the same visit to the health facility. OSS and AC models save opportunity costs by making the interactions shorter, as all services are provided in one place, which reduces the waiting time between different services in the health facility.

The total cost for DSDM and conventional care increased yearly, as the number of people enrolled in ART increased, following the country's efforts to offer treatment to all in need. The approach of including the number of people who would be receiving treatment in each model of care, accounting for retention outcome, resulted in the DSDMs having an increase in the total cost over time (beyond the cost resulting from the additional number of people in treatment) as they led to better retention and, consequently, more people on ART than the conventional care. On the other hand, the retention for conventional care is estimated to decrease over time and this is extensively discussed elsewhere [21].

Comparing our results to other studies in sub‐Saharan Africa is challenging because the costs are context‐driven and influenced by the characteristics of the models in each country. However, similar to what has been described, we found that some DSDMs are less and others more expensive than conventional care, depending on the services offered in each model, where they are offered, and when they are offered [4–7, 15, 16, 24].

Other challenges included a suboptimal reporting system that did not include clients in conventional care and information on which DSDMs were combined. Therefore, we assumed that only FT and 3MMD were combined and used other data sources and an exclusion method to calculate the percentage of enrolment in conventional care. Thus, clients not included in the reported models were assumed to be enrolled in conventional care, which includes clients enrolled in the new DSDMs being piloted in the country, such as 6 month and private pharmacy dispensing of antiretrovirals, which may explain the increase in enrolment in conventional care from 2020 to 2021, as these models’ pilots were being expanded in the country during this period [25, 26].

The first limitation was the simplicity of the model used to assess the economic impact of a complex infectious disease such as HIV and the short time horizon for the CEA, which did not allow us to measure the full benefit of the intervention. Therefore, our findings are an underestimation of the true benefit of the intervention for both the health system and its clients. The second limitation was estimating service interaction costs based on data available in the literature. Although we adjusted as much as possible to the Mozambique context, the results are not as accurate as they would be if estimated from the country's data. The third limitation was using the country's average wage to estimate the opportunity cost, intending to describe these costs for an average Mozambican using HIV services, while not all clients have an official employment. However, the findings from this study are useful in providing evidence on the benefit of implementing DSDMs in Mozambique compared with conventional care.

## 5 CONCLUSIONS

In conclusion, we found that DSDMs were more effective in retaining clients 12 months after ART initiation and less expensive to the health system and its clients than conventional care. Their implementation was estimated to have generated cost savings to the health system from 2022 to 2024.

## COMPETING INTERESTS

RB reports outside the submitted work, Regeneron Pharmaceuticals covered the cost of conference abstract and manuscript writing and service on a Gilead data monitoring board for which she is paid an honorarium.

## AUTHORS’ CONTRIBUTIONS

DAMU conceptualized the research question, the study design, the analytic strategy and conducted the analysis. MZ contributed substantially to the analytic strategy and reviewed the analysis. DAMU developed the first draft, and all authors provided substantial inputs, reviewed and approved the final version.

## FUNDING

The research reported in this publication was supported by the second edition of the complementary grant for doctoral activities for technicians of Instituto Nacional de Saúde (INS), offered by the government of Flanders, and by the Global Opportunities in Health Fellowship from the Department of Global Health, University of Washington. DAMU was supported by the Fogarty International Center of the National Institutes of Health under Award Number D43TW009343 and the University of California Global Health Institute. Publication costs were covered with funding contribution from the IAS—the International AIDS Society.

## DISCLAIMER

The content of this work is solely the responsibility of the authors and does not represent the funder's views.

## Supporting information

Supporting Information

## Data Availability

Data underlying the results reported in this article will be made available at the request of investigators whose proposed use of the data has been approved by an independent review committee identified for this purpose. Request submissions should be sent online at https://ins.gov.mz/institucional/unidade-organicas/direccoes/directora-de-inqueritos-e-observacao-de-saude/solicitacao-de-dados/, and a data access agreement will need to be assigned per the procedures of Instituto Nacional de Saúde.
