# Supplementary material for: Cost‐Effectiveness and Budget Impact Analysis of the Implementation of Differentiated Service Delivery Models for HIV Treatment in Mozambique: a Modelling Study
Source: J Int AIDS Soc. 2024 May 27;27(5):e26275. doi: 10.1002/jia2.26275 (PMC11129834; doi:10.1002/jia2.26275)
Supplement: Supplementary file 1 — Supporting Information [file JIA2-27-e26275-s001.docx]

**SUPPLEMENTAL MATERIAL**

**ANEXX I – Cost per model of care from the literature**

| **Model**  **of care** | **Cost per client per visit (USD)** | **Cost per client per year (USD)** | **Reference** | **Comment** |
| --- | --- | --- | --- | --- |
| Conventional Care | 18,04 | 374 | <https://onlinelibrary.wiley.com/doi/epdf/10.1111/tmi.12736> | Costs do not include ARVs and laboratory tests, but these costs are presented in the article $142 (ARVs), $10 (CD4), $50 (viral load) and $4 (creatinine) |
|  | - | 187 | <https://www.medrxiv.org/content/10.1101/2021.11.17.21266474v1> | Costs include ARVs ($164), clinic visits ($24) and viral load/laboratory tests ($6.90) |
|  | 5 | 152 | <https://bmchealthservres.biomedcentral.com/articles/10.1186/s12913-022-08629-4> | Costs include ARVs ($115.35), other medications ($9.99), visits ($5.00), laboratory tests ($13.04), local transportation costs, ongoing costs ($4.50), supervision, training, materials and management costs ($4.62) |
|  | 3,65 | 100 | <https://www.ncbi.nlm.nih.gov/pmc/articles/PMC7810408/#:~:text=Costs%20ranged%20from%20an%20annual,models%20(%24140%E2%80%93157).> | Costs include clinic visits ($4.99), pharmacy pickups ($4.32), laboratory tests ($4.61), ARVs ($87.96) |
|  | 5,58 | 23,36 | <https://www.ncbi.nlm.nih.gov/pmc/articles/PMC8035675/> | Costs include scheduled visits ($23.36), unscheduled visits ($2.55), viral load ($12.00), ARVs ($84.27) |
| Adherence Club | 16,52 | 300 | <https://onlinelibrary.wiley.com/doi/epdf/10.1111/tmi.12736> | Costs do not include ARVs and laboratory tests, but these costs are presented in the article $142 (ARVs), $10 (CD4), $50 (viral load) and $4 (creatinine) |
|  | 6,9 | 141 | <https://bmchealthservres.biomedcentral.com/articles/10.1186/s12913-022-08629-4> | Costs include ARVs ($96.88), other medications ($13.13), clinic visits ($6.90), laboratory tests ($14.85), local transportation costs, ongoing costs ($4.22), supervision, training, materials and management costs ($5.24) |
| Community Adherence Group | - | 178 | <https://www.medrxiv.org/content/10.1101/2021.11.17.21266474v1> | CAG plus quarterly dispensing of ARVs (CAG-3MC), includes ARVs ($163.45), visits ($9.23), laboratory tests ($6.43) |
|  | - | 167 | <https://www.medrxiv.org/content/10.1101/2021.11.17.21266474v1> | CAG with semi-annual dispensing of ARVs (CAG-6MC), includes ARVs ($161.08), clinic visits ($9.22), laboratory tests ($1.08) |
|  | - | 150 | <https://bmchealthservres.biomedcentral.com/articles/10.1186/s12913-022-08629-4> | Costs include ARVs ($103.20), other medications ($20.10), visits ($2.55), laboratory tests ($11.21), local transportation costs, ongoing costs ($7.40), supervision, training, materials and management costs ($5.44) |
|  | 0,99 | 130 | <https://www.ncbi.nlm.nih.gov/pmc/articles/PMC7810408/#:~:text=Costs%20ranged%20from%20an%20annual,models%20(%24140%E2%80%93157).> | Costs include clinic visits ($5.63), pharmacy pickups ($4.00), CAG meetings ($11.93), laboratory tests ($6.92), ARVs ($101.21) |
|  | 2.77 for 3 months | 114,2 | <https://www.ncbi.nlm.nih.gov/pmc/articles/PMC8035675/> | CAG with quarterly dispensing of ARVs (CAG-3MC) includes scheduled visits ($5.58), unscheduled visits ($0.20), CAG encounters ($13.59), viral load ($8.93), ARVs ($86.10) |
|  | - | 122,3 | <https://www.ncbi.nlm.nih.gov/pmc/articles/PMC5577715/> | Costs include service provision ($10.14): staff ($3.80), training ($2.08), supervision ($3.16), ongoing costs ($1.10); commodity costs ($112.16): ARVs ($94.45), laboratory tests ($12.47), opportunistic infections medications ($5.24) |
| Fast-track | - | 166 | <https://bmchealthservres.biomedcentral.com/articles/10.1186/s12913-022-08629-4> | Costs include ARVs ($133.96), other medications ($11.10), visits ($4.77), laboratory tests ($11.75), local transportation costs, ongoing costs ($2.10), supervision, training, materials and management costs ($2.79) |
|  | - | 120,73 | <https://www.ncbi.nlm.nih.gov/pmc/articles/PMC5577715/> | Costs include service provision ($8.57): staff ($3.81), training ($2.07), supervision ($0.49), ongoing costs ($2.20); commodity costs ($112.16): ARVs ($94.45), laboratory tests ($12.47), opportunistic infection medications ($5.24) |
| 3 multi-month dispensing of ARVs | - | 121,41 | <https://www.ncbi.nlm.nih.gov/pmc/articles/PMC5577715/> | Costs include service provision ($9.25): staff ($5.05), training ($2.0), ongoing costs ($2.20); commodity costs ($112.16): ARVs ($94.45), laboratory tests ($12.47), opportunistic infection medications ($5.24) |
| Family approach | Not found | Not found | NA | NA |
| One-stop shop TB | 74,04 | - | <https://www.ncbi.nlm.nih.gov/pmc/articles/PMC5886108/> | Costs include:  Total direct costs ($21.72): medical (any other facility -$1.71), patient travel (study clinic -$4.12, any other facility - $0.63); guardian travel (study clinic-$0.43; any other facility-$0.51); food (hospital-$0.26; special foods-$13.14); loan interest-$0.93 Total indirect costs ($52.34): patient income loss (job loss income loss-$15.40; care-seeking income loss-$30.45); opportunity costs of time (guardian: study clinic-$1.13; any other facility-$0.94; carer-$4.42) |
| One-stop shop MCH |  | From 220 to 1099 | <https://bmchealthservres.biomedcentral.com/articles/10.1186/s12913-019-3978-4> | Costs include:  Urban area (average $701/patient)- staff/salaries ($184); consumables ($157); equipment ($16); infrastructure ($324); training ($17)  Rural area (average - $302): personnel/salaries ($61); consumables ($127); equipment ($33); infrastructure ($79); training ($7) |
|  | - | 159,89 | <https://pubmed.ncbi.nlm.nih.gov/32726197/> | Costs include:  The mean cost per client-year on ART - $159.89 (range $100.91-$812.23): building ($2.62), equipment ($0.16), training ($2.03); personnel ($67.34), ART ($79.08), CPT prophylaxis ( $7.30); other supplies ($0.17); utilities and others ($1.20) The average cost of neonatal HIV care - $90.09 (range $41.53-$180.26): building ($1.35), equipment ($0.08), training ($1.60); personnel ($51.96), nevirapine ($1.62), DNA-PCR ($32.40); other supplies ($0.17); utilities and others ($0.91) |

**ANNEX II - Treatment Mix by year**

| Model of treatment | Reported number | Estimated number | Estimated percentage* | Reported number | Estimated number | Estimated percentage | Reported number | Estimated number | Estimated percentage |
| --- | --- | --- | --- | --- | --- | --- | --- | --- | --- |
| Total clients in ART | 1008146 | 1008146 | 100% | 1160903 | 1160903 | 100% | 1271105 | 1271105 | 100% |
| **Enrolled in reported DSD models*** | **-** | **437161** | **43,4 (100)** | **-** | **797499** | **68,7(100)** | **-** | **885219** | **69,6(100)** |
| Enrolled in 3MMD | 321668 | 118870 | 11,8(27,2) | 760821 | 257470 | 32,3(22,3) | 865654 | 245244 | 19,3(27,7) |
| Enrolled in FF | 227951 | 209615 | 20,8(47,9) | 433350 | 394542 | 49.5(34,0) | 529739 | 483281 | 38,0(54,6) |
| Enrolled in CAG | 88203 | 88203 | 8,8(20,2) | 103981 | 103981 | 13,0(9,0) | 107131 | 107131 | 8,4(12,1) |
| Enrolled in AC | 2137 | 2137 | 0,2(0,5) | 2698 | 2698 | 0,3(0,2) | 3105 | 3105 | 0,2(0,4) |
| Enrolled in FA | 18336 | 18336 | 1,8(4,2) | 38808 | 38808 | 4,9(3,3) | 46458 | 46458 | 3,7(5,2) |
| **Enrolled in DSD models not reported *** | **NA** | **570985** | **56,6 (100)** | **NA** | **363404** | **31,3(100)** | **NA** | **385886** | **30,4(100)** |
| OSS-MCH | NA | 54543 | 5,4(9,6) | NA | 106805 | 9,2 | NA | 113740 | 8,9(29,5) |
| OSS-TB | NA | 30846 | 3,1(5,4) | NA | 25027 | 2,2 | NA | 23155 | 1,9(6,0) |
| SOC | NA | 485596 | 48,1(85,0) | NA | 231572 | 19,9 | NA | 248991 | 19,6(64,5) |

* Reported or not in the AJUDA database

**In parathesis is the percentage among the models reported and the models not reported in the AJUDA database

**ANNEX III - Model and overall results by health care and societal perspectives per year**

1. 2019

| Decision | DSD model | Probability | Retention | Probability | Total probability | Payoff | | Population | | Health Care Cost  (Per person) | Health Care Cost (population) | Societal Cost  (Per person) | Societal  Cost (population) |
| --- | --- | --- | --- | --- | --- | --- | --- | --- | --- | --- | --- | --- | --- |
| Post-DSD | Family approach | 1,8% | Yes | 0,55 | 1,0% | 1 | 13395 | | $178,88 | | $2 396 131,62 | $217,28 | $2 910 506,93 |
| Post-DSD | Family approach | 1,8% | No | 0,45 | 0,8% | 0 | 10942 | | $89,44 | | $978 648,99 | $108,64 | $1 188 734,64 |
| Post-DSD | Fast flow + 3MMD | 11,8% | Yes | 0,55 | 6,5% | 1 | 86839 | | $120,42 | | $10 457 194,11 | $138,02 | $11 985566,61 |
| Post-DSD | Fast flow + 3MMD | 11,8% | No | 0,45 | 5,3% | 0 | 70935 | | $60,21 | | $4 271 018,48 | $69,01 | $4 895 249,72 |
| Post-DSD | Fast flow only | 20,8% | Yes | 0,55 | 11,4% | 1 | 153132 | | $129,38 | | $19 812 249,50 | $161,38 | $24 712 481,25 |
| Post-DSD | Fast flow only | 20,8% | No | 0,45 | 9,3% | 0 | 125087 | | $64,69 | | $8 091 891,85 | $80,69 | $10 093 287,27 |
| Post-DSD | CAG | 8,7% | Yes | 0,55 | 4,8% | 1 | 64436 | | $144,44 | | $9 307 115,87 | $165,24 | $10 647 381,80 |
| Post-DSD | CAG | 8,7% | No | 0,45 | 3,9% | 0 | 52635 | | $72,22 | | $3 801 293,49 | $82,62 | $4 348 696,60 |
| Post-DSD | Adherence club | 0,2% | Yes | 0,55 | 0,1% | 1 | 1561 | | $133,68 | | $208 696,54 | $152,88 | $238 670,91 |
| Post-DSD | Adherence club | 0,2% | No | 0,45 | 0,1% | 0 | 1275 | | $66,84 | | $85 237,66 | $76,44 | $97 480,05 |
| Post-DSD | 1-stop TB | 3,1% | Yes | 0,55 | 1,7% | 1 | 22534 | | $187,04 | | $4 214 806,41 | $244,64 | $5 512 779,30 |
| Post-DSD | 1-stop TB | 3,1% | No | 0,45 | 1,4% | 0 | 18407 | | $93,52 | | $1 721 448,02 | $122,32 | $2 251 577,44 |
| Post-DSD | 1-stop MCH | 5,4% | Yes | 0,55 | 3,0% | 1 | 39846 | | $187,04 | | $7 452 771,39 | $244,64 | $9 747 893,46 |
| Post-DSD | 1-stop MCH | 5,4% | No | 0,45 | 2,4% | 0 | 32548 | | $93,52 | | $3 043 925,93 | $122,32 | $3 981 319,72 |
| Post-DSD | Post-DSD SOC | 48,2% | Yes | 0,55 | 26,5% | 1 | 354748 | | $174,08 | | $61 754 450,68 | $250,88 | $88 999 061,27 |
| Post-DSD | Post-DSD SOC | 48,2% | No | 0,45 | 21,7% | 0 | 289778 | | $87,04 | | $25 222 291,70 | $125,44 | $36 349 773,33 |
|  |  |  |  |  |  |  | 1338100 | |  | | $162 819 172,24 |  | $217 960 460,28 |
| Pre-DSD | Pre-DSD SOC | 100% | Yes | 0,50 | 50,0% | 1 | 669236 | | $174,08 | | $116 500 643,16 | $250,88 | $167 897 985,74 |
| Pre-DSD | Pre-DSD SOC | 100% | No | 0,50 | 50,0% | 0 | 668864 | | $87,04 | | $58 217 902,42 | $125,44 | $83 902 271,13 |
|  |  |  |  |  |  |  | 1338100 | |  | | $174 718 546 |  | $251 800 256,87 |

1. 2020

| Decision | DSD model | Probability | Retention | Probability | Total probability | Payoff | Population | Health Care Cost  (Per person) | Health Care Cost (population) | Societal Cost  (Per person) | Societal Cost (population) |
| --- | --- | --- | --- | --- | --- | --- | --- | --- | --- | --- | --- |
| Post-DSD | Family approach | 3,3% | Yes | 0,64 | 2,1% | 1 | 30038 | $178,88 | $5 373 146,10 | $222,08 | $6 670 775,30 |
| Post-DSD | Family approach | 3,3% | No | 0,36 | 1,2% | 0 | 16860 | $89,44 | $1 507 968,04 | $111,04 | $1 872 146,37 |
| Post-DSD | Fast flow + 3M | 22,2% | Yes | 0,64 | 14,2% | 1 | 199284 | $120,42 | $23 997 768,38 | $140,22 | $27 943 589,78 |
| Post-DSD | Fast flow + 3M | 22,2% | No | 0,36 | 8,0% | 0 | 111858 | $60,21 | $6 734 949,53 | $70,11 | $7 842 340,33 |
| Post-DSD | Fast flow only | 34,0% | Yes | 0,64 | 21,8% | 1 | 305379 | $129,38 | $39 509 905,41 | $165,38 | $50 503 541,17 |
| Post-DSD | Fast flow only | 34,0% | No | 0,36 | 12,2% | 0 | 171408 | $64,69 | $11 088 415,17 | $82,69 | $14 173 767,97 |
| Post-DSD | GAAC | 9,0% | Yes | 0,64 | 5,7% | 1 | 80482 | $144,44 | $11 624 842,32 | $167,84 | $13 508 124,73 |
| Post-DSD | GAAC | 9,0% | No | 0,36 | 3,2% | 0 | 45174 | $72,22 | $3 262 500,29 | $83,92 | $3 791 041,60 |
| Post-DSD | Adherence club | 0,2% | Yes | 0,64 | 0,1% | 1 | 2088 | $133,68 | $279 160,51 | $155,28 | $324 267,24 |
| Post-DSD | Adherence club | 0,2% | No | 0,36 | 0,1% | 0 | 1172 | $66,84 | $78 346,12 | $77,64 | $91 005,27 |
| Post-DSD | 1-stop TB | 2,2% | Yes | 0,64 | 1,4% | 1 | 19371 | $187,04 | $3 623 171,48 | $251,84 | $4 878 419,08 |
| Post-DSD | 1-stop TB | 2,2% | No | 0,36 | 0,8% | 0 | 10873 | $93,52 | $1 016 839,43 | $125,92 | $1 369 123,40 |
| Post-DSD | 1-stop MCH | 9,2% | Yes | 0,64 | 5,9% | 1 | 82668 | $187,04 | $15 462 214,00 | $251,84 | $20 819 097,37 |
| Post-DSD | 1-stop MCH | 9,2% | No | 0,36 | 3,3% | 0 | 46401 | $93,52 | $4 339 454,79 | $125,92 | $5 842 858,71 |
| Post-DSD | Post-DSD SOC | 19,9% | Yes | 0,64 | 12,8% | 1 | 179239 | $174,08 | $31 201 863,02 | $260,48 | $46 688 081,80 |
| Post-DSD | Post-DSD SOC | 19,9% | No | 0,36 | 7,2% | 0 | 100606 | $87,04 | $8 756 771,44 | $130,24 | $13 102 963,14 |
|  |  |  |  |  |  |  | 1402902 |  | $167 857 316,00 |  | $219 421 143,27 |
| Pre-DSD | Pre-DSD SOC | 100% | Yes | 0,44 | 43,8% | 1 | 614597 | $174,08 | $106 989 068,31 | $260,48 | $160 090 260,31 |
| Pre-DSD | Pre-DSD SOC | 100% | No | 0,56 | 56,2% | 0 | 788305 | $87,04 | $68 614 055,92 | $130,24 | $102 668 826,33 |
|  |  |  |  |  |  |  | 1402902 |  | $175 603 124 |  | $262 759 087 |

1. 2021

| Decision | DSD model | Probability | Retention | Probability | Total probability | Payoff | Population | Health Care Cost  (Per person) | Health Care Cost (population) | Societal  Cost  (Per person) | Societal  Cost (population) |
| --- | --- | --- | --- | --- | --- | --- | --- | --- | --- | --- | --- |
| Post-DSD | Family approach | 3,7% | Yes | 69% | 2,5% | 1 | 38475 | $178,88 | $6 882 462,19 | $207,68 | $7 990 550,91 |
| Post-DSD | Family approach | 3,7% | No | 31% | 1,1% | 0 | 17649 | $89,44 | $1 578 516,80 | $103,84 | $1 832 660,83 |
| Post-DSD | Fast flow + 3M | 19,3% | Yes | 69% | 13,2% | 1 | 203105 | $120,42 | $24 457 865,48 | $133,62 | $27 138 847,24 |
| Post-DSD | Fast flow + 3M | 19,3% | No | 31% | 6,1% | 0 | 93166 | $60,21 | $5 609 497,08 | $66,81 | $6 224 389,63 |
| Post-DSD | Fast flow only | 38,0% | Yes | 69% | 26,1% | 1 | 400241 | $129,38 | $51 783 142,97 | $153,38 | $61 388 919,99 |
| Post-DSD | Fast flow only | 38,0% | No | 31% | 12,0% | 0 | 183593 | $64,69 | $11 876 645,14 | $76,69 | $14 079 763,73 |
| Post-DSD | GAAC | 8,4% | Yes | 69% | 5,8% | 1 | 88723 | $144,44 | $12 815 164,25 | $160,04 | $14 199 244,57 |
| Post-DSD | GAAC | 8,4% | No | 31% | 2,7% | 0 | 40698 | $72,22 | $2 939 202,79 | $80,02 | $3 256 646,46 |
| Post-DSD | Adherence club | 0,2% | Yes | 69% | 0,2% | 1 | 2571 | $133,68 | $343 755,44 | $148,08 | $380 784,75 |
| Post-DSD | Adherence club | 0,2% | No | 31% | 0,1% | 0 | 1180 | $66,84 | $78 841,51 | $74,04 | $87 334,32 |
| Post-DSD | 1-stop TB | 1,8% | Yes | 69% | 1,2% | 1 | 19176 | $187,04 | $3 586 747,61 | $230,24 | $4 415 166,64 |
| Post-DSD | 1-stop TB | 1,8% | No | 31% | 0,6% | 0 | 8796 | $93,52 | $822 633,12 | $115,12 | $1 012 633,93 |
| Post-DSD | 1-stop MCH | 8,9% | Yes | 69% | 6,1% | 1 | 94196 | $187,04 | $17 618 513,19 | $230,24 | $21 687 801,95 |
| Post-DSD | 1-stop MCH | 8,9% | No | 31% | 2,8% | 0 | 43209 | $93,52 | $4 040 867,69 | $115,12 | $4 974 173,31 |
| Post-DSD | Post-DSD SOC | 19,6% | Yes | 69% | 13,4% | 1 | 206208 | $174,08 | $35 896 661,98 | $231,68 | $47 774 233,96 |
| Post-DSD | Post-DSD SOC | 19,6% | No | 31% | 6,2% | 0 | 94589 | $87,04 | $8 233 025,11 | $115,84 | $10 957 187,83 |
|  |  |  |  |  |  |  | 1535575 |  | $188 563 542,33 |  | $227 400 340,06 |
| Pre-DSD | Pre-DSD SOC | 100% | Yes | 0,41 | 40,7% | 1 | 625079 | $174,08 | $108 813 774,80 | $231,68 | $144 818 332,64 |
| Pre-DSD | Pre-DSD SOC | 100% | No | 0,59 | 59,3% | 0 | 910496 | $87,04 | $79 249 560,60 | $115,84 | $105 471 841,68 |
|  |  |  |  |  |  |  | 1535575 |  | $188 063 335 |  | $250 290 174 |

**ANNEX IV – One-way sensitivity analysis.**

A. Cost difference comparing pre- and pos-DSD for base case scenario and the lower and higher values of the inputted parameters

|  | | Total post-DSD cost | Total pre-DSD cost | Cost difference |
| --- | --- | --- | --- | --- |
| Base scenario from health system perspective | | $173 463 476,86 | $179 461 668,41 | $5 998 191,55 |
| Base scenario from societal perspective | | $221 977 447,87 | $254 949 839,27 | $32 972 391,41 |
| SOC interactions | Lower value | $146 257 435,31 | $127 503 556,69 | $-18 753 878,62 |
|  | Higher value | $249 549 342,90 | $307 006 461,69 | $57 457 118,79 |
| AC interactions | Lower value | $171 818 296,41 | $179 461 668,41 | $7 643 371,99 |
|  | Higher value | $187 725 039,08 | $179 461 668,41 | $-8 263 370,68 |
| CAG interaction | Lower value | $171 963 102,94 | $179 461 668,41 | $7 498 565,46 |
|  | Higher value | $180 641 971,11 | $179 461 668,41 | $-1 180 302,70 |
| Start-up and training | Lower value | $173 425 130,19 | $179 461 668,41 | $6 036 538,21 |
|  | Higher value | $173 501 823,53 | $179 461 668,41 | $5 959 844,88 |
| ARVs | Lower value | $168 472 385,90 | $175 028 734,27 | $6 556 348,37 |
|  | Higher value | $178 454 567,82 | $183 894 602,54 | $5 440 034,72 |
| Lab test | Lower value | $166 122 212,18 | $172 863 812,95 | $6 741 600,77 |
|  | Higher value | $180 804 741,54 | $186 059 523,86 | $5 254 782,32 |
| Opportunity cost | Lower value | $197 720 462,36 | $217 205 753,84 | $19 485 291,48 |
|  | Higher value | $244 200 747,75 | $289 351 402,10 | $45 150 654,35 |

B. Lower and upper bound values of the inputted parameters for the tornado diagram

|  | | Health system perspective | Societal perspective |
| --- | --- | --- | --- |
| SOC interactions | Lower bound | $-24 752 070,17 | $-51 726 270,02 |
|  | Higher bound | $51 458 927,24 | $24 484 727,38 |
| AC interactions | Lower bound | $1 645 180,45 | $-25 329 019,41 |
|  | Higher bound | $-14 261 562,22 | $-41 235 762,08 |
| CAG interaction | Lower bound | $1 500 373,92 | $-25 473 825,94 |
|  | Higher bound | $-7 178 494,25 | $-34 152 694,11 |
| Start-up and training | Lower bound | $38 346,67 | $-26 935 853,19 |
|  | Higher bound | $-38 346,67 | $-27 012 546,53 |
| ARVs | Lower bound | $558 156,82 | $-26 416 043,03 |
|  | Higher bound | $-558 156,82 | $-27 532 356,68 |
| Lab test | Lower bound | $743 409,22 | $-26 230 790,64 |
|  | Higher bound | $-743 409,22 | $-27 717 609,08 |
| Opportunity cost | Lower bound | NA | $-13 487 099,93 |
|  | Higher bound | NA | $12 178 262,95 |
